# Genome Sequence of Pseudomonas sp. Strain LAP_36, A Rhizosphere Bacterium Isolated from King George Island, Antarctica

**DOI:** 10.1128/MRA.00731-21

**Published:** 2021-12-02

**Authors:** Sarah Mederos da Silveira, Sheila da Silva, Andrew Macrae, Rommel T. J. Ramos, Fabrício A. Araújo, Júnia Schultz, Aristóteles Góes-Neto, Mateus Matiuzzi da Costa, Siomar de Castro Soares, Vasco A. C. Azevedo, Flávia F. Aburjaile, Bertram Brenig, Alexandre S. Rosado, Selma Soares de Oliveira

**Affiliations:** a Laboratory of Molecular Microbial Ecology, Institute of Microbiology, Federal University of Rio de Janeiro, Rio de Janeiro, Brazil; b Laboratory of Genetics of Microorganisms Associated with Food and Industry, Institute of Microbiology, Federal University of Rio de Janeiro, Rio de Janeiro, Brazil; c Laboratory of Sustainable Biotechnology and Microbial Bioinformatics, Institute of Microbiology, Federal University of Rio de Janeiro, Rio de Janeiro, Brazil; d Laboratory of Biological Engineering, Science and Technology Park Guama, Belém, Pará, Brazil; e Laboratory of Molecular and Computational Biology of Fungi, Institute of Biological Sciences, Federal University of Minas Gerais, Belo Horizonte, Minas Gerais, Brazil; f Universidade do Vale do São Francisco, Petrolina, Pernambuco, Brazil; g Universidade Federal do Triângulo Mineiro—UFTM, Uberaba, Minas Gerais, Brazil; h Laboratory of Cellular and Molecular Genetics, Institute of Biological Sciences, Federal University of Minas Gerais, Belo Horizonte, Minas Gerais, Brazil; i Department of Molecular Biology of Livestock, Institute of Veterinary Medicine, Georg August University Göttingen, Göttingen, Germany; j Microbial Ecogenomics and Biotechnology Laboratory, Biological and Environmental Science and Engineering Division, King Abdullah University of Science and Technology, Thuwal, Makkah, Saudi Arabia; k Postgraduate Program in Plant Biotechnology and Bioprocesses, Decania, Health Sciences Center, Federal University of Rio de Janeiro, Rio de Janeiro, Brazil; Loyola University Chicago

## Abstract

Pseudomonas sp. strain LAP_36 was isolated from rhizosphere soil from Deschampsia antarctica on King George Island, South Shetland Islands, Antarctica. Here, we report on its draft genome sequence, which consists of 8,794,771 bp with 60.0% GC content and 8,011 protein-coding genes.

## ANNOUNCEMENT

The genus Pseudomonas comprises Gram-negative bacteria of the family *Pseudomonadaceae* (1). Pseudomonas are slightly curved rods, can be seen isolated or in pairs, are mobile with polar flagella (2, 3), and are widely distributed in nature, including Antarctic soil (4). Some strains produce biosurfactants, pigments, and bacteriocins of biotechnological interest (5, 6).

Pseudomonas sp. strain LAP26 was isolated from rhizosphere soil (pH 8.8) collected from Deschampsia antarctica at summer temperatures (2°C to 5°C) at Ullmann Point (62°05′015″S, 58°23′987″W), Admiralty Bay, King George Island, Antarctica. Rhizosphere soil (5 g) was added to 45 mL saline (0.85%). Serial 10-fold (1:10) dilutions were prepared, in which 0.1 mL of each dilution was spread over LB agar and incubated at 12°C for 4 weeks. A single colony was used to extract genomic DNA (gDNA) using the method of Ausubel and collaborators (7) and quantified using Qubit (Invitrogen) fluorimetry. The gDNA (5 μg/μL) was prepared using the NEBNext fast DNA fragmentation and library preparation kit (New England Biolabs, Inc.) and sequenced on an Illumina HiSeq 2500 platform (2 × 150 bp). FastQC v0.11.5 was used to verify the sequencing quality (8), and AdapterRemoval v2.3.0 (9) software was used for quality control. The estimated best k-mers were selected by KmerStream v1.1 (10), followed by assembly using Edena v3.131028 (11) and SPAdes v3.14.1 (12). Then, the results were combined, and CD-HIT v4.8.1 (PSI-CD-HIT) (13) was used to remove the redundant contigs and produce the final contig file. CheckM v1.1.3 was used to verify the completeness and contamination of the assembled genome (14). The genome was annotated using the PathoSystems Resource Integration Center (PATRIC) v3.6.9 (15) and the NCBI Prokaryotic Genome Annotation Pipeline (PGAP) v5.2 (16, 17). The strain was identified by analyzing the 16S rRNA coding sequence using the BLASTn v2.12.0 server (18) and genome-to-genome comparisons using the Average Nucleotide Identity (ANI) v3.8.3 and Tetra Correlation Search (TCS) v3.8.3 from the JSpeciesWS v3.8.3 server (19). ResFinder v2.1 (20) was used to verify the presence of resistance genes.

Sequencing resulted in 14,931,682 raw reads. The assembly resulted in 48 contigs comprising 6,053,373 bp, with 347× coverage, an *N*_50_ value of 790,041 bp, 60.00% GC content, 99.93% completeness, and 0.44% contamination (verified using CheckM). Using the PATRIC tool, 8,230 coding DNA sequences (CDS) and 88 RNAs were predicted, and PGAP annotation indicated 5,612 genes: 5,547 CDS, 5 rRNA genes, and 56 tRNA genes. No resistance genes were found using ResFinder. The 16S rRNA analysis resulted in 100% identity, 100% coverage, and an E value of 0.0 with Pseudomonas tritici strain SWRI145 (GenBank accession number CP077084.1) and 99.93% identity, 100% coverage, and an E value of 0.0 with Pseudomonas trivialis strain IHBB745 (CP011507.1). Using TCS resulted in a score of 0.99986 for Pseudomonas sp. strain 24E1. ANI, based on BLAST, (ANIb) yielded a score of 99.29 for Pseudomonas tritici strain SWRI145 (GCF_014268275.3) (21), a score of 94.66 for Pseudomonas trivialis IHBB745, and 85.51 for the type strain Pseudomonas trivialis DSM 14937. The strain has been classified as Pseudomonas sp. strain LAP_36. Unless otherwise noted, default parameters were used for all software tools.

### Data availability.

The whole-genome sequence of LAP_36 has been deposited at GenBank under accession number JAHZMS000000000.1, SRA accession number SRR16100360, BioProject accession number PRJNA647929, and BioSample accession number SAMN17068915.
